# Evolution and the duration of a doomed population

**DOI:** 10.1111/eva.12467

**Published:** 2017-03-17

**Authors:** Richard Gomulkiewicz, Stephen M. Krone, Christopher H. Remien

**Affiliations:** ^1^ School of Biological SciencesWashington State UniversityPullmanWAUSA; ^2^ Department of MathematicsUniversity of IdahoMoscowIDUSA

**Keywords:** conservation biology, continuous branching diffusion, environment change, evolutionary rescue, extinction, human pathogens, mutation, pest management

## Abstract

Many populations are doomed to extinction, but little is known about how evolution contributes to their longevity. We address this by modeling an asexual population consisting of genotypes whose abundances change independently according to a system of continuous branching diffusions. Each genotype is characterized by its initial abundance, growth rate, and reproductive variance. The latter two components determine the genotype's “risk function” which describes its per capita probability of extinction at any time. We derive the probability distribution of extinction times for a polymorphic population, which can be expressed in terms of genotypic risk functions. We use this to explore how spontaneous mutation, abrupt environmental change, or population supplementation and removal affect the time to extinction. Results suggest that evolution based on new mutations does little to alter the time to extinction. Abrupt environmental changes that affect all genotypes can have more substantial impact, but, curiously, a beneficial change does more to extend the lifetime of thriving than threatened populations of the same initial abundance. Our results can be used to design policies that meet specific conservation goals or management strategies that speed the elimination of agricultural pests or human pathogens.

## 1 Introduction

The persistence of populations is so fundamental to ecology and evolution that it is normally taken as given. Indeed, the central pursuits of those fields—understanding the distribution, abundances, and characteristics of species—would be completely moot without their persistence. In applied biology, however, the persistence of populations is often the paramount concern. Indeed, conservation biologists and wildlife managers seek ways to increase the duration of target species, and in pest and disease management in agricultural and medical settings, a main goal is to hasten the demise of pathogens.

It is well known that the duration of a species is largely determined by the capacities of its members to survive and reproduce and by its overall abundance (MacArthur & Wilson, 1967). Less is known about how inherent differences among individuals of a species impact the longevity of their populations, but much has been learned in recent years because of the growing number of empirical and theoretical studies investigating the combined demographic and evolutionary responses of species to environmental change (e.g., Bourne et al., 2014; Schiffers, Bourne, Lavergne, Thuiller, & Travis, 2013).

Among various research efforts connecting diversity and persistence, arguably the most developed is focused on the processes behind “evolutionary rescue” (reviewed in, e.g., Bell, 2012; Gonzalez, Ronce, Ferriere, & Hochberg, 2013). Evolutionary rescue refers to a declining population that is saved from extinction because of adaptive evolution. A still‐growing theoretical literature motivated by the topic allows considerable insight into a how a wide variety of genetic and ecological conditions factor into the persistence or extinction of populations (Baskett & Gomulkiewicz, 2011; Gomulkiewicz, Holt, Barfield, & Nuismer, 2010; Orr & Unckless, 2014; Ueker & Hermisson, 2016; Ueker, Otto, & Hermisson, 2014). In addition, a substantial number of experimental studies of evolutionary rescue have both tested components of the theory and explored additional mechanisms that might affect the probability of extinction or rescue (reviewed in Martin, Aguilée, Ramsayer, Kaltz, & Ronce, 2013; Carlson, Cunningham, & Westley, 2014; Alexander, Martin, Martin, & Bonhoeffer, 2014).

Evolutionary rescue is uncertain under most circumstances, and so the most useful theoretical studies predict probabilities of rescue for defined sets of genetic and demographic conditions. As rescue will fail unless genomes that allow population persistence exist or come into existence, these theoretical analyses consider both the extent to which these genomes occur and arise as well as the stochastic dynamics of their evolutionary spread. This theory, in effect, focuses exclusively on diametrically opposed *ultimate* outcomes: an adapting population that either goes extinct (failed rescue) or persists indefinitely (successful rescue).

In contrast to theory, experimental studies of evolutionary rescue are necessarily restricted to *finite* time horizons that may be far shorter than the duration of any given population headed to ultimate extinction. This practical limitation prompted Martin et al. (2013) to remark “Measuring the probability of rescue is not that straightforward. First, one must choose the time period over which populations can be said to be either doomed or rescued.” Similarly, conservation biologists and wildlife managers are often most interested in practical questions of how stressful conditions might affect the ability of natural populations to persist over prescribed periods of time (decade, century, etc.). In agricultural and medical settings, the main concern is predicting how pest management or disease treatment options might impact the time required to eradicate a target pathogen or malady.

In this theoretical study, we consider the antithesis of evolutionary rescue. That is, we seek a general understanding of how abundance and genetic diversity in fitness impact longevity of populations, including populations that necessarily are headed to extinction. We obtain analytical results by deriving the probability distribution of extinction times for a biologically simple stochastic model of population and evolutionary dynamics. Our analyses reveal not only the relative importance of a population's abundance and variation on its duration but also the extent to which genomes that are incapable of supporting permanent persistence nonetheless help slow a population's decline and thereby delay inevitable extinction.

## 2 Model of a genetically variable population at risk of extinction

Consider a polymorphic, asexually reproducing population with *G* genotypes (clones). We model the respective abundances of the clones, *X*_1_(*t*), *X*_2_(*t*), …, *X*_*G*_(*t*), at time *t* as *G* independent continuous branching (CB) diffusions (e.g., Lambert, 2006). Specifically, we assume the dynamics of genotype *i* are described by a CB diffusion with infinitesimal mean *a*_*i*_(*x*) = *r*_*i*_ *x* and infinitesimal variance *b*_*i*_(*x*) = *v*_*i*_ *x*, and that *X*_*i*_(*t*) ≥ 0 (Feller, 1951b). This is equivalent to the system of *G* stochastic differential equations

(1) $$dX_{i}=r_{i}X_{i}dt+\sqrt{v_{i}X_{i}}dW_{i},$$

*i* = 1, …, *G*, where *W*_1_, *W*_2_, …, *W*_*G*_ are independent standard Wiener processes. The parameter *r*_*i*_ is called the per capita growth rate or “Malthusian fitness” of genotype *i* and *v*_*i*_ is the per capita reproductive variance; *r*_*i*_ can be positive or negative, whereas *v*_*i*_ > 0.

This diffusion model provides a reasonable foundation to study how evolution affects extinction times for at least two reasons. First, the CB diffusion is a natural stochastic extension of density‐independent population growth and has been used extensively to model demographic stochasticity in population ecology (e.g., Lande, Engen, & Saether, 2003). Second, as a natural extension of discrete‐time branching processes, the CB diffusion has also been used in theoretical population genetics to study the fixation of genotypes in populations with stochastic changes in size (Feller, 1951a; Gillespie, 1974; Lambert, 2006; Martin et al., 2013). Note, however, that we here use the CB diffusion autonomously rather than as an explicit approximation of other stochastic models (we revisit the significance of this point in the Section 4).

The CB diffusion suffers a drawback that populations can grow without bound. Our focus, however, will be on populations that are small or in initial deterministic decline and thus are expected to be well below any carrying capacity or other population ceiling imposed by the environment. In those situations, the CB diffusion has been shown to provide a good approximation to the dynamics of populations well below carrying capacity (e.g., Goel & Richter‐Dyn, 1974; Parsons & Quince, 2007a, 2007b). This limitation will also be surmounted by our consideration of percentiles and conditional moments rather than simply unconditional moments of the probability distribution, which can be highly sensitive to relatively rare but extremely long persistence times.

## 3 Probability distribution of extinction time

### 3.1 Baseline: Genetically uniform populations

To establish a set of baseline results and expectations, we describe the persistence properties of a genetically uniform (“monomorphic”) population with initial density *X*(0) = *x* whose dynamics are described by the CB diffusion process (cf. Lambert, 2006)

(2) $$dX=rXdt+\sqrt{vX}dW.$$

Let *T* denote the time of extinction and write

(3) Φ(t;x,g)=P(T≤t∣X(0)=x),

for probability of extinction by time *t* given an initial population size *x*, where we introduce the shorthand *g* = (*r*, *v*) to identify a genotype with its two per capita parameters. Feller (1951a; see also Lambert, 2006) showed the following:

(4a) $$\Phi \left(t;\;x,\;g\right)={\left[f(t;\;g)\right]}^{x}$$

where *f*(*t*; *g*) is defined as follows:

(4b) $$\ln f\left(t;\;g\right)=\left\{\begin{array}{cc} -2r/\left[v\left(1-e^{-rt}\right)\right] & \text{if}\;r\neq 0\\ -2/(vt) & \text{if}\;r=0. \end{array}\right.$$

The mathematical form of (4a) is a manifestation of the branching property and, as 0 ≤ *f*(*t*; *g*) ≤ 1, the equation shows that the probability of extinction declines geometrically with initial density *x*.

**Figure 1:**
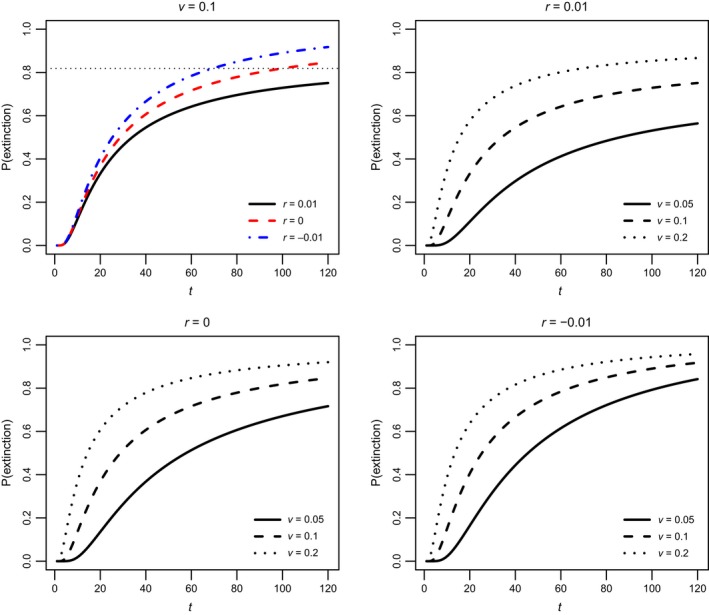
The per capita probability of extinction for genotype *g* = (*r*, *v*) as a function of time *t*. Each panel plots per capita risk functions *f*(*t*; *g*) for different combinations of per capita growth rate *r* and per capita reproductive variance *v* (Equation 4b). The horizontal dotted line in the upper left panel indicates the asymptotic per capita probability of extinction, *F*(*g*), for *r* = 0.01 (Equation (5)). Note that the corresponding probability of extinction for a population of density *x* that is monomorphic for genotype *g* is *f*(*t*; *g*)^*x*^

Note that the function *f*(*t*; *g*) defined by (4b) is independent of the initial density *x*. We will refer to it as the per capita risk function for genotype *g* because the extinction probability of a population of arbitrary size *x* that is genetically uniform for *g* can be found by raising *f*(*t*; *g*) to the power *x* (Equation 4a). Figure 1 shows example risk functions for genotypes with different combinations of *r* and *v*, representing growing, declining, and stationary monomorphic populations. Below we show that per capita extinction risk functions are useful for revealing effects of genetic variation on the duration of populations headed to extinction.

Let *F*(*g*) = lim_*t*→∞_ *f*(*t*; *g*) be the long‐term limit of the per capita risk function. We have from (4b)

(5) $$F\left(g\right)=\left\{\begin{array}{cc} 1 & \text{if}\;r\leq 0\\ \exp\;(-2r/v) & \text{if}\;r>0. \end{array}\right.$$

**Figure 2:**
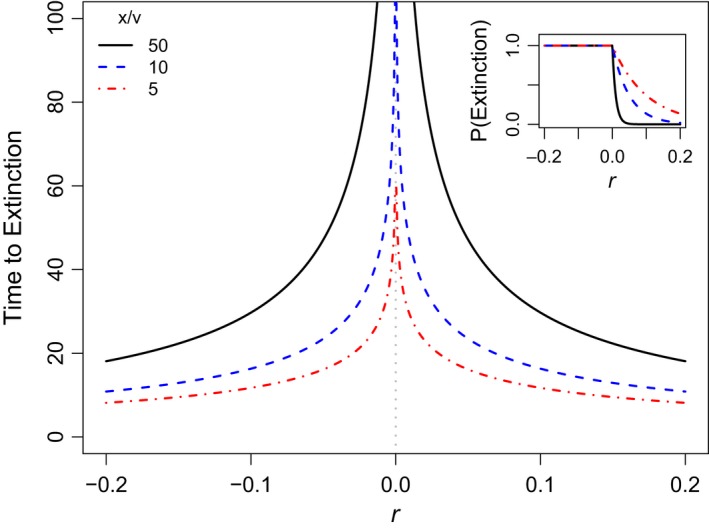
Mean (*r* ≤ 0) or conditional mean (*r* > 0) times to extinction (Equation 6b) for a population of density *x* that is genetically uniform for genotype *g* = (*r*, *v*), where *r* is the per capita growth rate and *v* is per capita reproductive variance. Note that both the mean and conditional mean times to extinction approach ∞ as the magnitude of *r* shrinks to zero. Comparing cases *x*/*v* = 5, 10, and 50 shows the effect of increasing density on the mean (or conditional mean) if *v* is assumed fixed or the effect of decreasing reproductive variance if assuming *x* is fixed. Inset: Probabilities of ultimate extinction *F*(*g*)^*x*^ where *g* = (*r*, *v*) (see Equation (5)) for the same parameter values

The asymptotic probability of extinction of a genetically uniform population with initial density *x* is *F*(*g*)^*x*^ (cf. Lambert, 2006). Clearly only genotypes with *r* > 0 have a nonzero chance of persisting indefinitely; all others face certain extinction (Figure 2 inset).

#### 3.1.1 Mean time to extinction

If a monomorphic population is necessarily headed to extinction (i.e., if *r* ≤ 0), the mean time to reach a critically low density *c* starting from *x* ≥ *c* can be derived using standard diffusion methods (Karlin & Taylor, 1981). Indeed, if *r* < 0, this mean time is

(6a) $$M_{c}\left(x,\;g\right)=\frac{\log x-\log c-e^{kc}E_{1}\left(kc\right)+e^{kx}E_{1}\left(kx\right)}{|r|}$$

where *k* = ∣ 2*r*/*v* ∣  and $E_{1}\left(a\right)=\int_{a}^{\infty }t^{-1}e^{-t}dt$ is the exponential integral function (Abramowitz & Stegun, 1972). Lande (1993) derived formula (6a) assuming a CB diffusion with reflecting upper boundary at *x* and *c* = 1. In the limit as *c* → 0

(6b) $$M\left(x,\;g\right)=\lim_{c\to 0}M_{c}\left(x,\;g\right)=\frac{\gamma +\log kx+e^{kx}E_{1}\left(kx\right)}{|r|}$$

where γ = 0.57721… is the Euler–Mascheroni constant (Figure 2). This, then, is the mean time to extinction for populations initially at density *x*.

Using (6b), it can be shown that for any *v* > 0, lim_∣*r*∣→0_ *M*(*x*, *g*) = ∞. This means that while populations with *r* = 0 are necessarily destined for extinction (see Equation (5)), it takes indefinitely long on average for them to disappear.

If *r* > 0, the mean time to extinction is infinite as a positive fraction of populations are certain to persist indefinitely. However, a substantial fraction of populations with *r* > 0 can go extinct (see Equation (5) and Figure 2 inset). Standard diffusion methods can be used to show that the mean time to extinction of populations with *r* > 0 conditional on extinction is given by expression (6b) (Figure 2). Indeed, it is possible to show that the CB diffusion with *r* > 0 but conditioned on extinction is itself a CB diffusion with *r* replaced by −*r* (e.g., Lambert, 2008). A direct consequence is that the conditional distribution of extinction times for *r* > 0 is given by (4a) with *r* replaced by −*r*.

The form of this conditional distribution has counter‐intuitive implications. For example, the expected lifetime of a genetically uniform population that is destined for extinction despite having positive *r* actually shrinks with increasing *r* (Figure 2). Intuitively, this is because a population with high *r* can only overcome its strong tendency to grow if it experiences a rapid succession of unfortunate reproductive outcomes. Of course, the probability of such misfortune declines exponentially with positive *r* (see Equation (5); Figure 2 inset).

Finally, note that (6b) serves as an upper bound to the average lifetime of an extinction‐bound population that is subject to density regulation. In fact, (6b) is an upper limit to the expected duration of the “final decline to extinction” of a population whose dynamics are described by a CB diffusion with *r* > 0 such that its densities are confined by a ceiling carrying capacity (Lande et al., 2003, pp. 47–49).

#### 3.1.2 Extinction time percentiles

The unconditional mean time to extinction is infinite for monomorphic populations with *r* ≥ 0. This allows only crude comparisons to populations with negative growth rates, which are expected to have finite expected durations (Equation 6b). By comparison, extinction time percentiles of the unconditional distribution—the time until there is a specified probability of extinction—allow for fine‐scale comparisons of populations with different per capita growth rates, regardless of sign. The probability distribution of extinction times (4) can be used to determine these percentiles.

Consider the amount of time it takes a population of density *x* to reach a specified probability *q* of extinction. This is the same as the time needed to reach per capita probability (or risk) of extinction equal to ρ = *q* ^1/*x*^. Let *t*(ρ; *g*) be the first time that the per capita risk of extinction is equal to ρ in a population monomorphic for genotype *g* = (*r*, *v*). By (4a), this is the solution *t* of *q* = ρ^*x*^ = Φ(*t*; *x*, *g*) or, equivalently, the solution *t* of

(7a) ρ=f(t;g).

**Figure 3:**
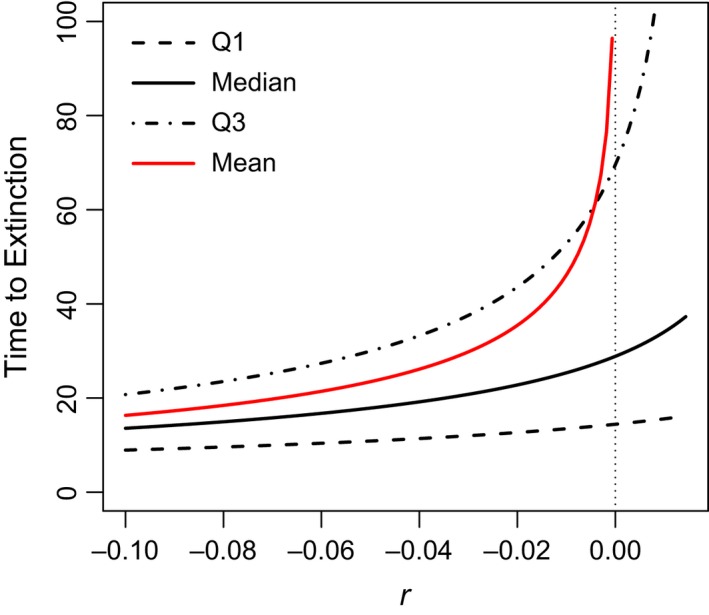
Per capita quartiles for the unconditional time to extinction [ *Q*_1_ = *t*(0.25; *g*),  *Median* = *t*(0.5; *g*),  *Q*_3_ = *t*(0.75; *g*); see (7)] for genotypes *g* = (*r*, *v*) with different intrinsic growth rates *r* and fixed per capita variance *v* = 0.1. The mean time to extinction (6b), shown for comparison to these percentiles, assumes *x*/*v* = 10

Inverting this using (4b) leads to the general solution

(7b) $$t\left(\rho ;\;g\right)=\left\{\begin{array}{cc} -\frac{1}{r}\log\left(1+\frac{2r}{v\log\rho }\right) & \text{if}\;r\neq 0\\ -\frac{2}{v\log\rho } & \text{if}\;r=0 \end{array}\right.$$

(Figure 3). As the maximum per capita probability of extinction of genotype *g* is *F*(*g*) (see Equation (5)), definition (7) makes sense only for values of ρ ≤ *F*(*g*), that is, only for genotypes *g* = (*r*, *v*) with *r* ≤ −(*v*/2) log ρ.

In contrast to the mean (6b), extinction time percentiles (7) are continuous at *r* = 0 (Figure 3). Moreover, for per capita extinction risks ρ well below *F*(*g*), the time to attain such risks (7b) rises slowly with *r* (see the median and first quartile functions in Figure 3). For a concrete comparison, consider two genotypes with the same reproductive variance (*v* = 1), one with potential for explosive growth (*r* = 0.2) and the other expected to decline catastrophically (*r* = −0.2). Despite this acute difference, the median time to extinction (time to risk ρ = 0.5) for the former genotype is a mere two time units longer than the latter. Put another way, if every genotype with *r* = −0.2 suddenly mutated to *r* = 0.2, the median time to extinction would increase only a minor amount. It seems reasonable to expect that the more realistic but comparatively slower process of adaptive substitution of such a remarkably beneficial mutation would provide even smaller advances in median longevity. Below, we show that this intuition is justified.

#### 3.1.3 Maladaptive landscape

**Figure 4:**
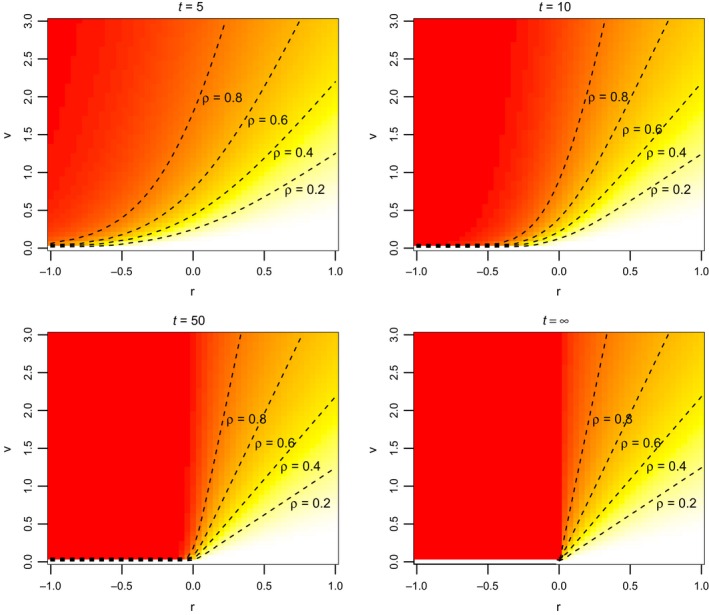
Maladaptive landscapes at different times *t*. Per capita risks of extinction for genotypes *g* = (*r*, *v*) are shown for each combination of *r* and *v*. Redder colors indicate genotypes with higher probabilities of extinction at the given time. Target per capita risk values ρ for each level curve of the per capita risk function *f*(*t*; *g*) are provided

The per capita risk function *f*(*t*; *g*) defined in (4b) is useful for comparing the vulnerabilities of different genotypes *g* = (*r*, *v*) to extinction. In comparing two monomorphic populations of the same initial density, the one consisting of the genotype with the higher value of *f*(*t*; *g*) is more likely to be extinct at time *t*. In this sense, *f*(*t*; *g*) can be used to construct an extinction risk‐based “maladaptive landscape” for a set of genotypes (Figure 4).

**Figure 5:**
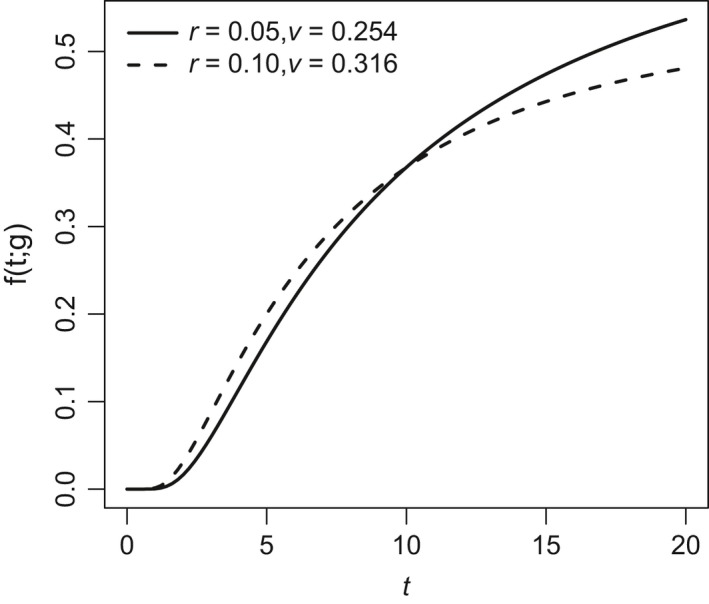
Per capita risk functions *f*(*t*; *g*) for two genotypes *g* = (*r*, *v*) that cross over time *t*. Solid curve: *r* = 0.05, *v* = 0.254. Dashed curve: *r* = 0.1, *v* = 0.316

Each level of the risk function describes an entire curve of genotypes whose combinations of *r* and *v* are equivalent with respect to extinction risk. These “neutral” curves tend to change with *t* (Figure 4). That is, two genotypes that are relatively neutral at one time may have distinct extinction risks at other times. Indeed, it is possible for one genotype to be more vulnerable to extinction than another for small *t* and less vulnerable for large *t* (Figure 5). For the example shown in Figure 5, the genotype with larger growth rate *r* but higher reproductive variance *v* (dashed curve) is more vulnerable to extinction than the genotype with lower *r* and *v* in the short term but less vulnerable in the long term. The genotypes have the same risk of extinction (i.e., are neutral) at time *t* ≈ 10.

With a baseline understanding of genetically uniform populations in hand, we turn to the process of extinction in polymorphic populations.

### 3.2 Genetically variable populations

Consider a population segregating for *G* distinct genotypes whose dynamics are described by the system of stochastic differential equations (1). Such a population reaches extinction at the random time *T* = *T*(ω) for a particular realization ω if the total population density $X\left(t,\;\omega \right)=\sum_{i=1}^{G}X_{i}\left(t,\;\omega \right)=0$ for *t* ≥ *T*(ω) but *X*(*t*, ω) > 0 for *t* < *T*(ω).

We aim to understand how the probability distribution of *T* over different realizations depends on the growth rates, reproductive variances, and initial densities of the *G* genotypes. Let **x** = (*x*_1_, *x*_2_, …, *x*_*G*_) denote their initial densities and **g** = (*g*_1_, *g*_2_, …, *g*_*G*_) where we extend the genotype notation *g*_*i*_ = (*r*_*i*_, *v*_*i*_) for *i* = 1, …, *G*. Define

(8) Φ(t;x,g)=P(T≤t∣X(0)=x),

where **X**(*t*) = (*X*_1_(*t*), *X*_2_(*t*), …, *X*_*G*_(*t*)) are the densities of the genotypes at time *t*. Assuming that the genotypes are independent implies that (using Equation 4)

(9) $$\Phi \left(t;\;x,\;g\right)=\prod_{i=1}^{G}\Phi \left(t;\;x_{i},g_{i}\right)=\prod_{i=1}^{G}{\left[f(t;\;g_{i})\right]}^{x_{i}}$$

where *f*(*t*; *g*_*i*_) is given by (4b) with *g*_*i*_ = (*r*_*i*_, *v*_*i*_).

Equation (9) can be rewritten in a simpler, more intuitive form as follows. Let

(10) $$x=\sum_{i=1}^{G}x_{i}$$

be the total initial density of the population and

(11) $$p_{i}=x_{i}/x$$

be the initial frequency of genotype *i* = 1, …, *G*. Then, (9) is equivalent to

(12a) $$\Phi \left(t;\;x,\;g\right)={\left[\tilde{f}\left(t;\;g\right)\right]}^{x}$$

where

(12b) $$\tilde{f}\left(t;\;g\right)=\prod_{i=1}^{G}{\left[f(t;\;g_{i})\right]}^{p_{i}}$$

is the geometric mean per capita risk function. Note the formal similarity of (12a) to (4a).

As we saw above, percentiles are expedient for comparing the extinction times of monomorphic populations. With that in mind, we will employ percentiles to characterize extinction times for genetically variable populations. From (12), the first time that the probability of extinction is equal to *q* for a polymorphic population with initial genotypic densities **x** = (*x*_1_, *x*_2_, …, *x*_*G*_) is defined as the implicit solution *t* = *t*(ρ, **g**) of

(13) $$\tilde{f}\left(t;\;g\right)=\rho ,$$

where ρ = *q* ^1/*x*^ is the target per capita risk of extinction for a population of total density *x* (Equation (10)).

It is possible to derive an approximate formula for *t*(ρ, **g**) for a population that is expected to grow or decline slowly. If |*r*_*i*_| ≪ 1 for all segregating genotypes *i*, then, from (4b), log *f*(*t*; *g*_*i*_) ≈ −2/(*v*_*i*_ *t*). Substituting this in (13) and solving for *t* lead to the approximation

(14) $$t\left(\rho ,\;g\right)\approx -\frac{2}{{\overset{¯}{v}}_{H}\log\rho }$$

where ${\overset{¯}{v}}_{H}={\left(\sum_{i=1}^{G}p_{i}/v_{i}\right)}^{-1}$ is the harmonic mean reproductive variance. Expression (14) reveals that in populations with slow rates of growth or decline, the time needed to reach any specified probability of extinction is, to first approximation, independent of *r* and inversely proportional to the overall variation in reproduction, ${\overset{¯}{v}}_{H}$. Note that this approximation for *t*(ρ, **g**) is bounded below by −2/(*v*_max_ log ρ) and above by −2/(*v*_min_ log ρ), where *v*_min_ and *v*_max_ are, respectively, the minimum and maximum of *v*_1_, *v*_2_, …, *v*_*G*_. As harmonic means disproportionately weight the contributions of smaller values, the longevity of a population with slow expected change will be extended most strongly by genotypes with lower reproductive variance.

The results we obtained above for polymorphic populations account for both evolutionary and population dynamic processes and combine the distinct influences of the abundances, frequencies, and demographic parameters (in essence, the fitnesses) of genotypes on the time to extinction. In the sections that follow, we use our formulas to investigate the consequences of replacing, adding, or removing genotypes, as well as sudden environmental change on the longevity of a population.

#### 3.2.1 Mutation/replacement

Understanding how evolution impacts the duration of a doomed population is a main motivation for this study. Because total abundance per se has a direct influence on population persistence, a conceptually clear way to separate out the influence of evolution on time to extinction is to compare genetically variable and genetically uniform populations with the same initial density. This raises the question, however, of what monomorphic population would provide the most biologically informative comparison. Spontaneous mutation presents a clear option as, in essence, it causes a change in genotype with no change in density at the moment it arises. This suggests that a meaningful comparison would be to contrast a genetically uniform population in which a mutation appears spontaneously with the same ancestral population sans mutation. Although we refer below to mutation, our results also apply to a deliberate replacement of individuals by the same density of a different genotype, as in the experiments of Hufbauer et al. (2015).

Consider a mutation or substitute genotype with parameters g* = (r*, v*) that appears in an ancestral population with density *x* that is monomorphic for genotype *g* = (*r*, *v*). Let x* denote the abundance of the mutant genotype when it first arises. We assume the mutation occurs at time *t* = 0.

To use the extinction time probability distribution (9) for *t* > 0, note that there are just two genotypes—ancestral and mutant—so *G* = 2. We then set *x*_1_ = *x* − x*, *r*_1_ = *r*,* v*_1_ = *v* for the ancestral genotype and *x*_2_ = x*, *r*_2_ = r*, *v*_2_ = v* for the mutant in (9). With these substitutions, the extinction time distribution for the population that includes the mutation, which we denote by Φ*(*t*; *x*, *g*), is

(15a) $$\Phi^{*}\left(t;\;x,\;g\right)={\left[f(t;\;g)\right]}^{\left(x-x^{*}\right)}{\left[f(t;\;g^{*})\right]}^{x^{*}}$$

(15b) $$\Phi^{*}\left(t;\;x,\;g\right)=\Phi \left(t;\;x,\;g\right){\left[\frac{f(t;\;g^{*})}{f(t;\;g)}\right]}^{x^{*}},$$

where Φ(*t*; *x*, *g*) is the extinction time distribution (4a) of the original population sans mutation.

Rearranging (15b) suggests an intuitive measure for the effect of mutation on the extinction profile of the original population:

(16) $${\left[\frac{\Phi^{*}\left(t;\;x,\;g\right)}{\Phi (t;\;x,\;g)}\right]}^{1/x^{*}}=\frac{f(t;\;g^{*})}{f(t;\;g)}.$$

The left‐hand side of (16) is the relative change in extinction risk per mutant at time *t*, which is equal to the ratio of mutant‐to‐ancestral per capita risk functions.

**Figure 6:**
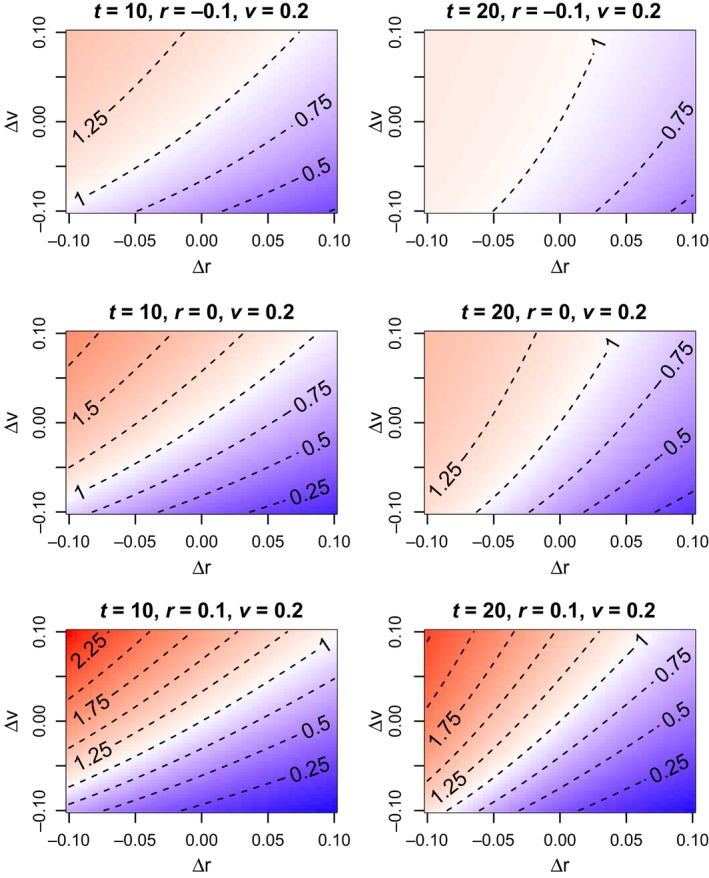
Ratios of mutant‐to‐ancestral genotypic per capita risk functions, *f*(*t*; g*)/*f*(*t*; *g*), at times *t* = 10 (left column) and *t* = 20 (right column) assuming the mutant's respective growth rate and reproductive variance are r* = *r* + Δ*r* and v* = 0.2 + Δ*v*, where *r* = −0.1, 0, 0.1 and *v* = 0.2 are the ancestral values. Dashed contours correspond to indicated ratio values. Warmer (colder) colors show mutants that increase (decrease) the risk of extinction compared to the ancestral genotype replaced. The increased intensity of color from top to bottom panels shows mutant effects are greatest on ancestral backgrounds with the lowest risk of extinction

As (15b) shows, the risk function ratio (16) predicts how a mutation will impact the probability of extinction at time *t* of the population in which it arose. In accord with intuition, a mutant with risk function value *f*(*t*; g*) below or above that of the ancestral genotype, *f*(*t*; *g*), will reduce or increase, respectively, the risk of extinction. Figure 6 shows how the mutant‐to‐ancestral per capita risk ratio (16) varies over mutant genotypes that arise in ancestral populations with negative, zero, and positive growth rates, *r*.

**Figure 7:**
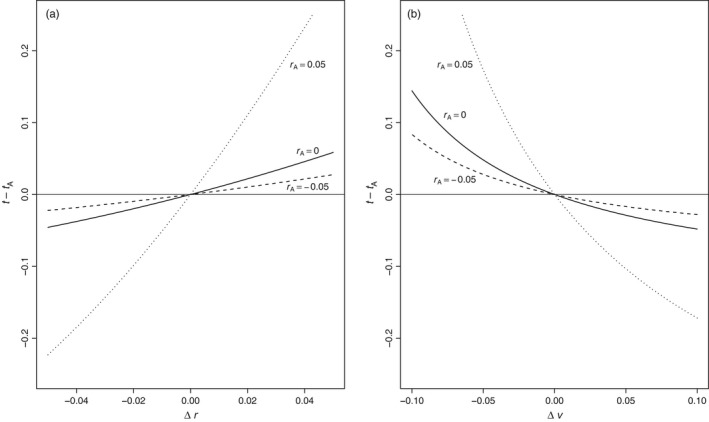
Changes in per capita median time to extinction caused by mutations in *r* (left panel) or in *v* (right panel) that arise in declining, stationary, and growing monomorphic ancestral populations as indicated by *r*_*A*_, the ancestral per capita growth rate. The mutant parameters are r* = *r*_*A*_ + Δ*r* and v* = *v*_*A*_ + Δ*v*. The time to per capita extinction probability ρ = 0.5 is *t*_*A*_ in populations without and *t* in populations with the mutation. Both panels assume ancestral reproductive variance *v*_*A*_ = 0.2 and mutation initial frequency p* = 0.01

Another way to understand the evolutionary impact of a spontaneous mutation is in terms of the relative impact it has on the duration of the population in which it arises. Using (13), the time t* after the mutation event needed to reach probability *q* of extinction is defined implicitly by

(17) $$\rho =f\left(t^{*};\;g\right){\left[\frac{f(t^{*};\;g^{*})}{f(t^{*};\;g)}\right]}^{p^{*}}$$

where p* = x*/*x* is the initial frequency of the mutation and ρ = *q* ^1/*x*^ is the target per capita risk of extinction in a population of size *x*. Although it does not allow a closed‐form expression, (17) can be solved numerically for t* given any combination of parameters and initial conditions. Figure 7 indicates how mutations in the growth rate *r* or in the reproductive variance *v* impact per capita median times to extinction of declining, stationary, and growing monomorphic ancestral populations. The results suggest that mutations have the least impact on populations that are expected to decline (i.e., *r* negative) and greatest impact on populations with positive expected growth rates. Even then, the evolutionary effect of de novo mutation on longevity is remarkably small—well less than a time unit in absolute terms.

If the effects of the mutation on *r* and *v* are small, then an approximate expression for its impact on duration compared to no mutation can be derived using Taylor series. Let *t* be the time (7) to per capita extinction risk ρ in the absence of mutation and define Δ*t* = t* − *t*, Δ*r* = r* − *r*, and Δ*v* = v* − *v*. Then, the relative impact of mutation on the duration of the population is approximately (Appendix A)

(18) $$\Delta t\approx p^{*}\left(\frac{e^{rt}-1-rt}{r^{2}}\Delta r-\frac{e^{rt}-1}{rv}\Delta v\right).$$

As most mutations are initially rare, p* < < 1 and so approximation (18) suggests that the impact of mutation on duration should be proportionately small. This agrees with numerical results based on (17) shown in Figure 7 which assume p* = 0.01. In contrast to new mutations, genotypic replacements may involve values of p* that are substantially above zero and thus could have significant impacts on longevity.

The coefficients of mutational effects Δ*r* and Δ*v* in (18) are, respectively, always positive and always negative. Both coefficients depend on *r*, but the coefficient of Δ*r* does not depend on *v*. This suggests a greater role for ancestral *r* than *v* in shaping the impacts of mutation on extinction time. Moreover, the magnitudes of both coefficients increase exponentially with *r*. These features are borne out in Figure 7. Contrary perhaps to conventional wisdom, this implies that evolution is generally least impactful in harsh environments where population growth rates are below zero.

#### 3.2.2 Abrupt environmental change

Sudden environmental change is an important driver of evolutionary diversification in natural populations (e.g., Estes & Arnold, 2007; Franks, Sim, & Weis, 2007) and a central concern in applied settings ranging from conservation biology (e.g., rapid climate change, toxic spills, habitat destruction and restoration) to wildlife management (species relocation programs) to agricultural systems (pesticide application) to human health (e.g., antimicrobial treatment). Our results can be used to project how a sudden change in environment could affect the time to extinction of genetically diverse populations.

Consider a one‐time change in the environment that affects the abundances or demographic properties of all genotypes present. Suppose that before the environmental change the genotype with fitness parameters *g*_*i*_ = (*r*_*i*_, *v*_*i*_) has density *x*_*i*_ and that these change suddenly to $g_{i}^{\prime }=\left(r_{i}^{\prime },\;v_{i}^{\prime }\right)$ and $x_{i}^{\prime }$, respectively. If we set the time of the abrupt change to *t* = 0, then, using (4), the probability distribution of extinction times for *t* ≥ 0 after the change is Φ(*t*; **x** ^′^, **g** ^′^), where $x^{\prime }=\left(x_{1}^{\prime },\;\;x_{2}^{\prime },\ldots ,\;\;x_{G}^{\prime }\right)$ and $g^{\prime }=\left(g_{1}^{\prime },\;\;g_{2}^{\prime },\ldots ,\;\;g_{G}^{\prime }\right)$.

**Figure 8:**
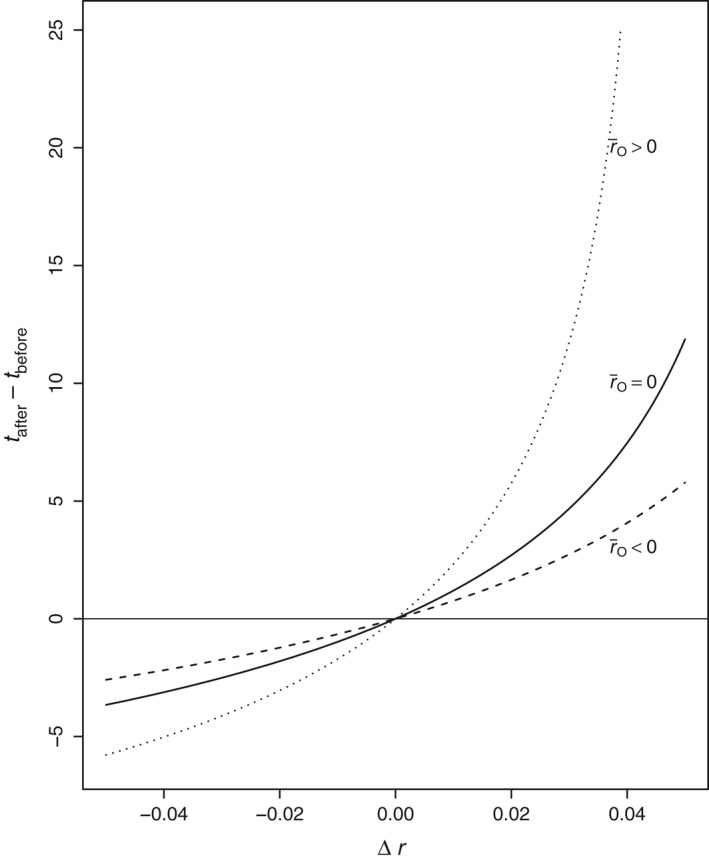
Effect on time to extinction of an abrupt shift in environment. *t*_before_ and *t*_after_ are the respective per capita median times to extinction under environmental conditions before and after the shift. All curves assume two equally abundant genotypes with the same reproductive variance *v* = 0.2 but different intrinsic growth rates (*r*_1_, *r*_2_), corresponding to mean intrinsic growth rate ${\overset{¯}{r}}_{O}=0.5r_{1}+0.5r_{2}$ in the original environment. The abscissa, Δ*r*, is a one‐time change in *r* experienced identically by both genotypes, that is, $r_{1}^{\prime }=r_{1}+\Delta r$ and $r_{2}^{\prime }=r_{2}+\Delta r$. Other parameters are ${\overset{¯}{r}}_{O}>0$: (*r*_1_, *r*_2_) = (0.05, 0); ${\overset{¯}{r}}_{O}=0$: (*r*_1_, *r*_2_) = (−0.025, 0.025); ${\overset{¯}{r}}_{O}<0$: (*r*_1_, *r*_2_) = (0, −0.5)

Figure 8 illustrates the impact of an abrupt environmental change that induces identical modifications in the growth rates of two equally frequent genotypes (*G* = 2) without affecting their reproductive variances or abundances. The average growth rate of the population just prior to the change is zero in the left panel and positive in the right. Comparing the figure panels suggests that the impact of environmental change on the time to extinction is greater for the population with higher average growth rate. We observed a similar association for spontaneous mutations (Figure 7). An implication of this for conservation biology is that habitat restoration would help most to prolong the lifetime of those species that are the least threatened; the persistence of those same species, however, would be most sensitive to environmental degradation.

This example, like the mutation section just above, highlights consequences of a discrete change in a population that leaves its total abundance unmodified. We now explore how changes in the densities of some or all genotypes can affect the lifetime of a population.

#### 3.2.3 Supplementation and removal

In applications ranging from species preservation and restoration to pest and disease eradication, population genetic diversity is often altered by adding or eliminating genotypes. This changes both the relative frequencies of genotypes and—in contrast to spontaneous mutation and genotype replacement—the total population density. The results above show that genetic diversity and abundance can contribute separately to the duration of a population. Here, we consider their combined effects on time to extinction in the two most common contexts in applied population biology: supplementation and removal.

##### Supplementation

Suppose a manager wants to preserve a population that is currently headed to extinction. If habitat restoration is not an option (see Section 3.2.2), then the manager could still aid the threatened population by adding densities **y** = (*y*_1_, *y*_2_, …, *y*_*G*_) of *G* different strains to achieve a particular conservation goal, say, to ensure a maximum probability *q* of extinction at future time *t* > 0. The scenario corresponds to managing a threatened population with current extinction outlook

(19) P(T≤t∣X(0)=x)>q.

The management goal requires additions **y** that lower the extinction probability to *q* at time *t*. Stated in terms of our modeling framework (8) and (9), these densities are solutions **y** of

(20) $$q=\Phi \left(t;\;x+y,\;g\right)=\prod_{i=1}^{G}{\left[f(t;\;g_{i})\right]}^{x_{i}+y_{i}}$$

where *q*, *t*, and the initial densities **x** are fixed. That is, supplements **y** must satisfy

(21a) $$\sum_{i=1}^{G}y_{i}\log f\left(t;\;g_{i}\right)=-S\left(q,\;t,\;x\right)$$

where

(21b) $$S\left(q,\;t,\;x\right)=\sum_{i=1}^{G}x_{i}\log f\left(t;\;g_{i}\right)-\log q$$

describes the shortfall that management efforts must overcome to achieve the conservation goal. The status quo (19) implies *S*(*q*, *t*; **x**) > 0.

It is possible to achieve the management goal using any single genotype (*g*_*j*_, say) by adding

(22) $$y_{j}=y_{j}^{+}:=-\frac{S(q,\;t,\;x)}{\log f(t;\;g_{j})},$$

of the genotype and none of the rest (i.e., *y*_*i*_ = 0 for *i* ≠ *j*). The general equation (21) that any **y** must satisfy can be recast in terms of these “pure supplements”:

(23) $$\sum_{i=1}^{G}\frac{y_{i}}{y_{i}^{+}}=1.$$

Each term of the sum in (23) is bound between 0 and 1 because $0\leq y_{i}\leq y_{i}^{+}$ (if $y_{i}>y_{i}^{+}$, the management goal is surpassed).

Although any solution **y** of (21) could be used to meet the management goal, some supplementation strategies may be more efficient to implement than others. The smallest supplement consistent with the conservation goal is achieved using a pure strategy (22) utilizing a genotype with the minimum value among $y_{1}^{+},\ldots ,\;y_{G}^{+}$. Note from definitions (4b) and (22) that this genotype has the lowest per capita risk among all genotypes at time *t*. A pure strategy is also best if supplementation costs differ among genotypes. Indeed, suppose the per capita cost of using genotype *g*_*i*_ is *c*_*i*_, then the management goal could be achieved most economically by supplementing exclusively with a genotype that minimizes the total cost $c_{i}y_{i}^{+}$ among the *G* genotypes.

In many and probably most conservation settings, managers would need to draw supplements from a source population for which the parameters *r* and *v* of individual genotypes are impractical to assess. If the distribution of genotypes in the source is unknown or partly known, Equation (21) can be used to develop a management policy as follows. Suppose the true frequency of genotype *g*_*i*_ in the source pool is *π*_*i*_ ($\sum_{i=1}^{G}\pi_{i}=1$). What size random sample from this pool would ensure a probability *p* of meeting the management goal? If the true frequencies are known, and population sizes are sufficiently large to invoke the strong law of large numbers, randomly sampling a total density *y* from this pool will result in densities *y*_*i*_ = *yπ*_*i*_ of genotype *i* ∊ {1, …, *G*}. By Equation (23), the sample density necessary to meet the management goal (20) with probability *p* = 1 is

(24) $$y={\left[\sum_{i=1}^{G}\frac{\pi_{i}}{y_{i}^{+}}\right]}^{-1},$$

which is the harmonic mean of the pure supplement densities defined in (22). If the exact values of the *π*_*i*_ are not known but it is possible to find constants *a*_*i*_ such that *P*(*a*_1_ ≤ *π*_1_, …, *a*_*G*_ ≤ *π*_*G*_) ≥ *p*, then the management goal can be met with probability *p* by choosing a suitably enlarged sample size $\hat{y}$ that satisfies

(25) $$\hat{y}={\left[\sum_{i=1}^{G}\frac{a_{i}}{y_{i}^{+}}\right]}^{-1}.$$

More generally, linear programming methods (e.g., Gill, Murray, & Wright, 1981) based on (23) can be used to find management solutions **y** that are optimal given other practicalities (e.g., a genotype whose availability for supplementation is limited).

##### Removal

The primary aim of pest and disease management is to speed the demise of pathogens or, equivalently, elevate the probability of their extinction at any time. Eradication goals are often attained by administering drugs, pesticides, or other treatments that reduce growth rates of all pathogens. The impacts of sublethal options can be assessed by applying approaches described in Section 3.2.2. We examine here how lethal treatments or removal protocols that instantly reduce pathogen or pest densities affect the persistence of pathogen populations.

Consider the densities of pathogen genotypes that would need to be removed to achieve a desired probability of elimination *q* within a time frame *t*. Intervention is needed only if *q* > Φ(*t*; **x**, **g**), the probability of pathogen clearance within that time without treatment (see Equation 12). Let *z*_*i*_ be the density of genotype *g*_*i*_ to be removed. Clearly, the removal densities are restricted to 0 ≤ *z*_*i*_ ≤ *x*_*i*_ for all *i*. Letting ***z*** = (*z*_1_, …, *z*_*G*_) and solving Φ(*t*; **x** − **z**, **g**) = *q* for **z** shows that the removal densities must satisfy

(26) $$\sum_{i=1}^{G}\frac{z_{i}}{z_{i}^{-}}=1$$

where $z_{i}^{-}=S\left(q,\;t,\;x\right)/\log\left(\right.f\left(t;\;g_{i}\right)$; *S*(*q*, *t*, **x**) is defined in (21b). The requirement *q* > Φ(*t*; **x**, **g**) ensures that $z_{i}^{-}>0$ for all genotypes.

In contrast to supplementation, it may be impossible to achieve the treatment objective using a “pure” removal strategy, **z** = (0, …, 0, *z*_*j*_, 0, …, 0). This would occur whenever $x_{i}<z_{i}^{-}$ for all *G* genotypes. Standard methods of linear programming (e.g., Gill et al., 1981), however, can be applied to identify efficient removal strategies. For example, the strategy requiring the smallest overall density of pathogens to be cleared can be computed by minimizing *z* = *z*_1_ + *z*_2_ + ··· + *z*_*G*_ subject to the linear constraints (26) and 0 ≤ *z*_*i*_ ≤ *x*_*i*_ for *i* = 1, …, *G*.

## 4 Discussion

Evolutionary rescue studies establish, both theoretically and empirically, that adaptive evolution can enable the indefinite persistence of populations that would otherwise go extinct. By extension, it stands to reason that evolution might also delay the demise of populations whose extinction it cannot prevent altogether. Our findings show, however, that evolution does little to extend the lifespan of populations headed to extinction.

That evolution does little to prolong the remaining lifetime of populations headed to extinction was previously predicted in Gomulkiewicz and Holt (1995). Their speculation, however, was based on an informal inspection of results generated by deterministic models of population and evolutionary dynamics and use of a positive density as ersatz extinction. Our study now rigorously establishes the veracity of this conjecture, accounting fully for stochastic population and evolutionary dynamics as well as exact extinction. We found that evolution is equally impotent in delaying the final demise of populations that are expected to decline as it is of populations expected to increase but that nonetheless descend to extinction through a series of unfortunate chance events.

We appraised the impact of evolution by comparing the extinction time of a monomorphic, mutant‐free “ancestral” population to that of a descendant population of the same density but polymorphic due to the spontaneous appearance of a mutation. Our numerical and analytical results (see Equation (18)) show that the impact of the mutation on persistence time is proportional to its initial frequency, which suggests that the ineffectiveness of evolution in this assessment is due primarily to the scarcity of new mutations.

Of course, populations often harbor substantial amounts of standing genetic variation, and it is natural to ask how evolution impacts longevity in those populations. Our results apply to these populations with ample genetic diversity as we did not require assumptions about the rarity or commonness of genotypes. Isolating the role of evolution is less clear, as it is not obvious which comparable nonevolving population would make for an appropriate comparison. Although it is easy to concoct possibilities (e.g., a genetically monomorphic population of size *x* with *r* and *v* values equal to the respective means of the polymorphic population), it is unclear what, if any, biological insights such highly artificial comparisons might provide. In contrast, comparing populations with and without a mutant has clear biological relevance as it addresses a classic question about the impact of a new mutation, albeit in a novel context. While it may be difficult to separate the role of evolution meaningfully from other processes such as population dynamics, our methods explicitly account for evolution (adaptive and otherwise) and can be used to forecast and explain population longevity in a variety of contexts, including species’ responses to environmental change, conservation and wildlife biology, the management of agricultural pests, and the treatment of pathogens relevant to health and disease.

Our analyses revealed some unexpected results. First, conditioned on extinction, the mean time to extinction increases as the magnitude of *r*, the intrinsic rate of population growth, shrinks. This is intuitive when *r* is negative: Populations expected to decline more slowly should persist longer. But the result holds even when *r* is positive, which means that among populations expected to increase, those with the higher growth rates go extinct more rapidly, on average. Conditioning on extinction is key to making sense of this anomalous‐sounding result as populations with high expected growth rates can go extinct only if they “escape” the strong tendency to grow by experiencing a rapid succession of bad demographic luck (mathematically, the process conditioned on extinction has a negative growth rate, viz. −*r*). Although the conditional time to extinction is reduced, the probability that a population goes extinct declines exponentially with *r* (Figure 2, inset).

A second unusual finding concerns the relationship between the harshness of the initial environment and the impact of adaptive evolution on time to extinction. Conventional wisdom holds that selection is stronger in harsh than in benign demographic conditions. To the contrary, our analyses of spontaneous changes of fixed magnitude to the environment or to the genetic composition of a population (e.g., new mutations with fixed change in genotype) demonstrate that adaptive evolution can have less impact—good or bad—on struggling populations (lower *r*) than on thriving populations (higher *r*). The limited impact of further negative changes on a threatened population might be understandable as the time to extinction is already short and obviously cannot be negative. In contrast, the upside potential is boundless, yet the time to extinction is nearly the same with or without beneficial changes (Figure 3). It is, alas, not intuitively clear to us why the same beneficial changes extend the time to extinction more when they appear in populations with higher than with lower expected growth rates. In contrast to spontaneous changes of fixed magnitude (e.g., new mutations that change *r* or *v* by a fixed amount), replacement of individuals by genotypes sampled from a fixed distribution (as in experiments of Hufbauer et al. (2015)) could potentially affect longevity more in harsh demographic conditions than in benign ones.

Our analyses demonstrate the central importance of per capita extinction risk functions for understanding the remaining lifetimes of both genetically uniform and polymorphic populations. Much the same way as fitness functions are used in population genetics to describe the adaptive spread and relative prevalence of genes and genomes within populations, per capita risk functions describe the relative impacts of different genotypes on the time to extinction of populations. In this sense, per capita risk functions serve as “misfitness” functions.

We found that a change in the expected growth rate (*r*) has more of an impact on extinction time than a comparably sized change in reproductive uncertainty (*v*). This asymmetry can be traced to the double role that *r* plays. Whereas *r* and *v* make comparable contributions to the probabilities of extinction in the short term, *r* alone determines whether or not a population is certain to go extinct in the long run and so in effect sets the stage for the extinction.

The theory developed here helps enrich our understanding of recent experimental studies that examined impacts of genetic variation and abundance on persistence in stressful environments (Hufbauer et al., 2015; Ramsayer, Kaltz, & Hochberg, 2013). Those experiments, like our model, show a direct link between abundance and persistence and also confirm the potential impact of genetic variation on persistence. Although both studies were designed with a focus on successful cases of evolutionary rescue, visual inspection of trajectories that were recorded for failed populations (fig. 1 in Ramsayer et al. (2013) and fig. 2 in Hufbauer et al. (2015)) shows that monomorphic and polymorphic replicates with the same initial size have similar extinction times, which matches our predictions.

Our theory also helps extend and refine interpretations of patterns in studies with treatments that manipulated genetic diversity while controlling for initial population size. Ramsayer et al. (2013) found that genetically “diversified” populations of the Gram‐negative bacterium *Pseudomonas fluorescens* were more likely to persist when exposed to the antibiotic streptomycin than same‐sized populations started with a single clone. The diversified populations initially harbored higher frequencies of resistant mutants than the clonal populations. Similarly, the “genetic rescue” treatment in Hufbauer et al. (2015) replaced a portion of flour beetles (*Tribolium castaneum*) from a stock population with substitutes obtained from a separate population that were partially pre‐adapted to a stressful food resource (corn meal). Persistence on a corn meal diet of the genetically mixed population was compared to that of a stock population with the same starting size. The genetically variable populations in both studies were more able to persist for the duration of the experiment than the less variable controls. The flour beetle manipulation also relieved inbreeding depression by fostering production of more fit hybrid offspring. The experimental treatments thus introduced novel genotypes that had lower risks of extinction than the original genotypes they replaced or descended from. In the vernacular of our model, the diversified and genetic rescue populations were relatively successful because *f*(*t*; *g*_new_) < *f*(*t*; *g*_original_). Of course, the hybrid genotypes in the *Tribolium* experiments were ephemeral and so their impact on persistence is not given complete account in our analyses, which assume asexual reproduction. Theory showing how sexual reproduction, including inbreeding, hybridization, and genetic recombination, affects time to extinction we leave to a future study.

The models we analyzed here assume not only a genetically simple type of inheritance but also make ecologically simplistic assumptions about population dynamics and environmental change. Perhaps most glaring is the absence of density dependence in population growth rates. This assumption in particular is a considerable benefit for analysis because it implies that individuals and genotypes can be tracked independently which in turn allows application of the extensive mathematical theory developed for branching processes. Although the independent branching assumption precludes analysis of extinction times for populations with density‐dependent dynamics, it might be possible to derive tractable results using approaches allowing interdependent per capita growth rates that have been developed to analyze evolutionary rescue (e.g., Lambert, 2008; Ueker et al., 2014). Note too that some of our results—such as the expected time to extinction—provide upper bounds for populations with ceiling density dependence. Finally, we considered the impacts of a single abrupt environmental change on the duration of populations. Our results could be extended to scenarios that assume gradual environmental change by imagining a discrete sequence of small abrupt changes or by extending approaches (such as those of Lynch and Lande (1993) and Bürger and Lynch (1995)) that model continuous environmental change directly.

Similar to evolutionary rescue theory, our extinction time formulas depend on two essential demographic parameters: *r*, the per capita growth rate (or “Malthusian fitness”) and *v* the per capita reproductive variance. Methods for estimating these parameters from population time series are discussed in Ramsayer et al. (2013), Martin et al. (2013), and Alexander et al. (2014). In microbial cells, for example, it is possible to estimate *r* and *v* by measuring the birth and death rates of cells: If *b* and *d* are per capita birth and death rates, then *r* = *b* − *d* and *v* = *b* + *d* (e.g., Martin et al., 2013). Note that our extinction time probability distributions can be written as parametric versions of the risk and hazard functions that are central to survival analysis, which is a well‐developed collection of statistical methods that are used to evaluate stochastic time‐to‐event data (e.g., Klein & Moeschberger, 2013). Survival analysis may be particularly useful in analyzing experimental data because experiments can consider only finite time horizons in practice and so the ultimate fate of any population that remains at the end of an experiment is equivocal. We plan to develop the statistical connection between our models and survival analysis in a future publication.

Besides the demographic parameters, many of our results and those for evolutionary rescue depend on a population's initial density, *x*. We have taken care to avoid using “count” and “number” to describe *x* as those terms imply a discrete scale for abundances, whereas CB diffusions assume a continuous scale for population size. Our use of the CB diffusion as a stand‐alone stochastic modeling framework thus has the drawback that there is no obvious value on the continuous scale that corresponds to one individual of a specific genotype, which is the initial count of a single new mutant. Nonetheless, some experiments measure population size as a density (e.g., Ramsayer et al., 2013) and it is often possible to use a CB diffusion to approximate a discrete‐scale branching process by prudently rescaling population number as a density (for details and examples see Goel & Richter‐Dyn, 1974; Lambert, 2006). Regardless of scale, “per capita” has the usual meaning (i.e., “per unit abundance”) and, of course, extinction corresponds to a density of zero.

The formulas we derived here can be used not only to interpret data on temporal patterns of extinction but also to forecast population longevity. In addition, our methods can be used to design management strategies to meet specific conservation or wildlife policy goals (Lankau, Jørgensen, Harris, & Sih, 2011; Nicotra, Beever, Robertson, Hofmann, & O'Leary, 2015; Pierson et al., 2014; Smith, Kinnison, Strauss, Fuller, & Carroll, 2014) and to assess efforts aimed at speeding the eradication of agricultural pests or medical pathogens (Alexander et al., 2014; Wu, Saddler, Valckenborgh, & Tanaka, 2014). These strategies include manipulations of overall population abundance, genetic diversity, or both. We have also shown how our formulas can be extended to compare the costs and benefits of different management or treatment plans and to design economical schemes. Even if evolution has relatively little natural impact on how long a population has until its demise, as our results suggest, our findings demonstrate that well‐designed managed changes have significant potential to lengthen or shorten the lifetime of a population.

## Appendix A

### A.1

If *t* is defined by (7) and *t** is the solution of (17) then

(27) $$\log f\left(t;\;g\right)=\left(1-p^{*}\right)\log f\left(t^{*};\;g\right)+p^{*}\log f\left(t^{*};\;g^{*}\right)$$

or, equivalently,

(28) $$\left[\ln f\left(t^{*};\;g\right)-\ln f\left(t;\;g\right)\right]-p^{*}\left[\ln f\left(t^{*};\;g\right)-\ln f\left(t^{*};\;g^{*}\right)\right]=0.$$

Using first‐order Taylor expansions for the left‐hand side, (28) is approximately

(29) $$\frac{\partial \ln f(t;\;g)}{\partial t}\Delta t+p^{*}\left[\frac{\partial \ln f(t;\;g)}{\partial r}\Delta r+\frac{\partial \ln f(t;\;g)}{\partial v}\Delta v\right]\approx 0,$$

where Δ*t* = *t** − *t*, Δ*r* = *r** − *r*, and Δ*v* = *v** − *v*. By definition (4b) of *f*(*t*; *g*) we have

(30a) $$\frac{\partial \ln f(t;\;g)}{\partial t}=Cr^{2},$$

(30b) $$\frac{\partial \ln f(t;\;g)}{\partial r}=-C\left(e^{rt}-1-rt\right),$$

(30c) $$\frac{\partial \ln f(t;\;g)}{\partial v}=-C\frac{r(e^{rt}-1)}{v},$$

where

(30d) $$C=\frac{e^{rt}}{v{\left(e^{rt}-1\right)}^{2}.}$$

Substituting expressions (30) into (29) and solving for Δ*t* gives the approximation (18).
